# Mosque as a Vaccination Site for Ethnic Minority in Kanagawa, Japan: Leaving No One Behind Amid the COVID-19 Pandemic

**DOI:** 10.1017/dmp.2022.78

**Published:** 2022-03-23

**Authors:** Hitomu Kotani, Hirofumi Okai, Mari Tamura

**Affiliations:** 1 Department of Urban Management, Graduate School of Engineering, Kyoto University, Kyoto, Japan; 2 Faculty of International Social Studies, Kyoai Gakuen University, Gunma, Japan; 3 Department of International Studies, Graduate School of Frontier Sciences, The University of Tokyo, Chiba, Japan

**Keywords:** coronavirus, vaccine, Muslims, Islam, disaster

## Abstract

Ethnic minorities with different languages and religions are potentially vulnerable not only during natural hazard-related disasters, but also during the coronavirus disease 2019 (COVID-19) pandemic. Their vaccination coverage may be lower, and vaccination strategies should prevent them from being left behind. This report presents the first case in Japan where a mosque, being the hub of foreign Muslims, was used as a vaccination site from the end of July 2021. The targeted mosque was Ebina Mosque in Kanagawa Prefecture, and most of the vaccine recipients were foreign Muslims. The mosque differed from other vaccination sites in that reservations could be made easily through the managers, and linguistic diversity (i.e., the mosque managers and mosque-related volunteers served as interpreters) and gender were considered. These efforts are likely to have removed some barriers to vaccination for ethnic minorities and contributed to “no one will be left behind.”

Vaccination against coronavirus disease 2019 (COVID-19) is an important measure against the pandemic. However, as is the case with natural hazard-related disasters, where ethnic minority groups tend to be left behind due to language and religious differences,^1,2^ there are concerns that vaccination rates among them may be lower.^3,4^ Vaccination strategies to avoid exclusions, both for humanitarian reasons and for overall social safety, should be implemented.

Ethnic minorities in Japan are likely to be excluded from the vaccination process. To get vaccinated, people first require vaccination vouchers distributed by local governments. Then, people have three main vaccination options: (1) individual vaccination (at local hospitals or clinics), (2) group vaccination (at local gymnasiums or convention centers set up by local governments), and (3) workplace vaccination (arranged by their companies or universities). Those ineligible for workplace vaccination should make an appointment for individual or group vaccination through their local government’s booking website. However, information on vaccination vouchers and booking websites is primarily written in Japanese. There are barriers before making a booking and accessing a vaccination site for people with limited Japanese language skills and who are unfamiliar with the Japanese medical system. Furthermore, few vaccination sites are multilingual, or culturally or religiously sensitive, which is considered a heavy psychological burden.

This report presents a case study of group vaccination at Ebina Mosque in Ebina City, Kanagawa Prefecture, Japan, that was initiated to reduce these hurdles. It is extremely rare for mosques, being the bases of an ethnic minority group, to be used as vaccination sites in Japan. As far as we know, as of October 1, 2021, Ebina Mosque and Osaka Islamic Center are the only mosques in Japan used as vaccination sites, and Ebina Mosque was the first and a large-scale case. Our report describes how vaccination was conducted at the mosque and how ethnic minorities were considered. Our description is based on information obtained through two surveys: (1) a field survey on September 18, 2021 (observations at the site and interviews with a deputy representative of the mosque, an *imam* (i.e., a religious leader), and a city office worker); and (2) an email survey to the city office (i.e., the local authority) before and after the field survey.

## Ebina Mosque as a Vaccination Site

Ebina Mosque is a place of worship used daily by Muslims—mainly people with foreign citizenship. Even amid the COVID-19 pandemic, on weekdays, 10 to 50 Muslims attend each of the five daily prayers; on Fridays, 300 to 400 Muslims gather for mass prayers. The nationalities of the worshippers are diverse: while a majority are Sri Lankans, some are Pakistanis, Bangladeshis, Indians, Fijians, and so forth. They not only live in Ebina City, but also in neighboring municipalities. The four-story mosque, newly built in 1998, has worship space on the first through third floors. It is managed and operated by a Sri Lankan representative, a deputy Pakistani representative, a Sri Lankan imam, and a Pakistani imam. It is one of 19 mosques managed by the religious corporation, Darussalam.^5^

Vaccination at this mosque started on Saturday, July 31, 2021. The Ebina City Office had three group vaccination sites (i.e., two gymnasiums and one convention center). However, from late June 2021, the City Office planned to use the mosque where foreign residents regularly gathered, as a vaccination site. The City Office intended to combat the inability of people to receive vaccinations due to language barriers, and also to increase the vaccination rate in the region as a whole. After lobbying the mosque, the City Office executed the plan. (The current city mayor was from the area where the mosque was located, and in building the mosque, he mediated between local Muslims and Japanese residents. Such a relationship might have backed this vaccination drive, but future research is required to identify the key factors.)

Vaccinations occurred every Saturday and continued until October 23, 2021. Consequently, approximately 750 people associated with the mosque were vaccinated. The target population was Ebina residents first, but it was expanded to residents of neighboring municipalities. Most of the targeted people were foreign Muslims who regularly used the mosque, but a few Japanese (i.e., wives and children of foreign Muslims) and people of other religions who had a connection to the mosque were also included. Reservations were accepted at the mosque, not through the city’s booking website, and were reported to the City Office by the mosque managers. Each week, approximately 100 people were vaccinated for 1.5 h, starting at 2:00 pm, by doctors, nurses, and paramedics entrusted by the City Office. Pfizer vaccines were used, and a 75-m^2^ room on the second and/or third floor was used as the vaccination site.

**Figure 1.**
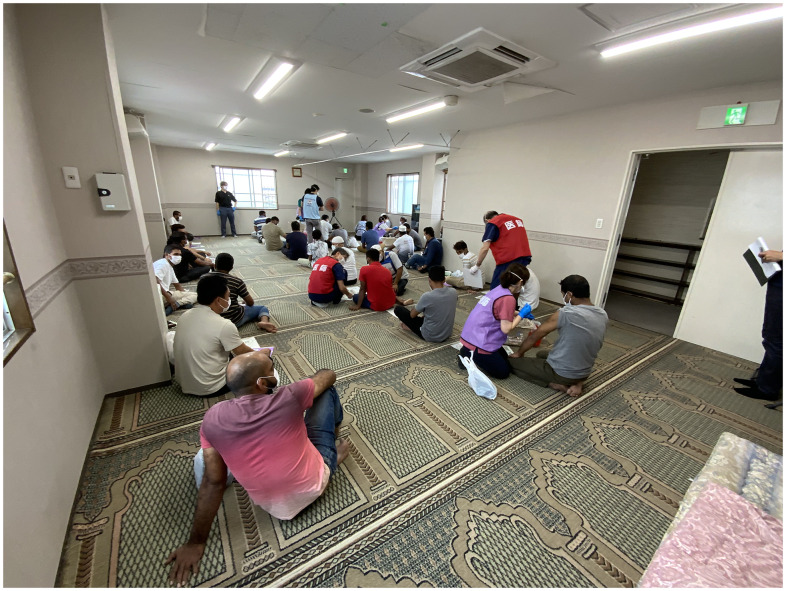
People receiving vaccinations on the third floor of Ebina Mosque (doctors, nurses, and paramedics wore scrimmage vests).

On the vaccination day, the reception desk opened at 1:30 pm. The residence card and the certificate of residence of each person with a reservation were checked at the desk. Once checked, people went to the designated vaccination rooms on the second or third floor, which were well ventilated with open windows and an electric fan. They entered the rooms in order and sat down to wait. When approximately 30 people had gathered, a Japanese doctor explained the precautions in Japanese in front of them. After that, doctors walked around and conducted individual medical interviews. Thereafter, nurses and paramedics administered vaccinations to those who had completed the medical interview (Figure 1). After vaccination, the vaccine recipients waited for 15 min for observation. Japanese traditional bedding (a *futon*) prepared by the mosque was placed at the back of the room in case anyone became unwell. Those whose waiting time had expired left the room. This cycle was repeated until the scheduled number of people for the day was completed.

There were two notable aspects of vaccination at the mosque: multilingual support and gender support. For multilingual support, medical questionnaires in Japanese and English were available. Confirmation and questions about vaccination precautions (i.e., history of anaphylaxis) in Japanese, English, Tamil, Urdu, Sinhalese, and Bengali were also posted at the reception. Furthermore, three or four interpreters conversant in several foreign languages were on standby. They could interpret Japanese into not only English but also Bengali, Tamil, Sinhala, and other languages. The interpreters were all volunteers from among the daily worshippers as well as the mosque representative and imams. They interpreted beside the doctors during the initial group explanations and the individual medical interviews. Additionally, vaccine recipients were given stickers to wear, naming the language in which they were fluent (Japanese, English, Sinhalese, Tamil, and so on), enabling Japanese staff to easily determine the appropriate interpreter required by the recipients.

Support regarding gender was based on the fact that most of the vaccine recipients were Muslims. Measures were taken to avoid females sharing the same space with males. Specifically, the vaccination rooms were separated for women and men (i.e., women on the second floor and men on the third floor), or if they shared the same room, the women’s space was separated by a curtain. Female nurses administered vaccinations to female recipients.

## Discussion

This study reported the first case in Japan where a mosque regularly used by foreigners was used as a group vaccination site for the COVID-19 vaccine. The foreigners were familiar with the mosque, they could make an appointment through those they had a relationship with, and they could receive multilingual and gender-related support on site. These facts are likely to have enabled the following: (1) an increase in their motivation to get vaccinated and (2) closing the intention-behavioral gap through increasing accessibility, availability, and convenience.^3,6^ From these perspectives, Ebina Mosque as a vaccination site probably promoted the vaccination of ethnic minorities, so that they were not left behind. It is estimated that there are approximately 180,000 foreign Muslims in Japan as of 2018,^7^ being overwhelmingly small compared with Japan’s total population (i.e., approximately 130 million). As of 2017, there are approximately 100 mosques in Japan, operated mainly by foreign Muslims.^5^ This case study recommends that vaccination strategies should take advantage of their community bases or networks^8^ in a Muslim-minority society.

Additionally, mosques in Japan are beginning to be known for approaching not only Japanese but also foreign residents with specific needs, providing appropriate support during the COVID-19 crisis^9^ and natural hazard-related disasters (e.g., earthquakes).^10–13^ The government and health-care sectors should be encouraged to work closely with mosques to realize disaster management plans that avoid exclusions.

Globally, other countries and regions experience similar issues. Religious or faith-based organizations are also expected to work actively in times of disaster.^14,15^ Hopefully, this case study will be useful to these areas, helping to realize a world where no one is left behind.
